# Factors associated with medication non-adherence among patients with severe mental disorder - A cross sectional study in a tertiary care centre

**DOI:** 10.1016/j.rcsop.2022.100178

**Published:** 2022-09-12

**Authors:** Papiya Ghosh, Sivaprakash Balasundaram, Avudaiappan Sankaran, Vigneshvar Chandrasekaran, Sukanto Sarkar, Sunayana Choudhury

**Affiliations:** a Department of Psychiatry, Mahatma Gandhi Medical College & Research Institute, Sri Balaji Vidyapeeth (Deemed – to – be) university, Puducherry, India; b Department of Psychiatry, All India Institute of Medical Sciences (AIIMS), Kalyani, India

**Keywords:** Medication adherence, Stigma, Psychotropics, Attitude, Caregiver, Mental disorders

## Abstract

**Background:**

Medications are an essential treatment modality of mental disorders. There is limited scientific literature on medication non-adherence among patients in Severe Mental Disorders with respect to patient-related factors. The current study explores the factors associated with medication non-adherence in such patients. Objectives: To study the relationship between socio-demographic, clinical, treatment related factors, self-stigma, patients' & caregivers' attitude towards psychotropic medications, doctor-patient communication and medication non-adherence in patients with severe mental disorders.

**Methods:**

A cross-sectional observation study, where patients with severe mental disorders attending Psychiatry Outpatient services, and their caregivers, were recruited. Sociodemographic and clinical variables were recorded using data collection form and illness-specific severity scales. Patients were administered Medication adherence rating scale (MARS), Internalised stigma of mental illness inventory (ISMI- 9), Attitude of patients towards psychotropic medication scale and Doctor-patient communication questionnaire (DPCQ). The Attitude of caregivers towards psychotropic medication scale was administered for caregivers.

**Results:**

Among 152 patients, 58 (38.16%) patients had a diagnosis of schizophrenia, 11 (7.24%) had delusional disorder, 32 (21.05%) patients were diagnosed with bipolar disorder type 1 – mania and 19 (12.50) with bipolar disorder type 2 – depression whereas 32 (21.05%) had major depressive disorder. Majority of the patients were non-adherent to medications (88.16%). MARS score was significantly higher among patients of middle socioeconomic class (*p* = 0.014), urban domicile (*p* = 0.033) and those with higher caregivers' age (*p* = 0.019) . Among 79.61% of patients, ISMI-9 score was minimal to mild. Most patients (76.97%) and caregivers (83.55%) had negative attitude towards psychotropic medications. MARS score negatively correlated with BPRS (*p* = 0.0001*), HAM-D (*p* = 0.0004*), YMRS (*p* = 0.0007*), ISMI-9 (*p* ≤0.0001*) and the attitude of the caregivers towards psychotropic medicationsnegative scale scores (*p* = 0.003*) . MARS score positively correlated with DPCQ scores (*p* ≤0.0001*) .

**Conclusion:**

Medication adherence was higher among patients belonging to uppermiddle socioeconomic status, urban domicile and higher caregiver age. Higher severity of illness, self-stigma and negative attitude of patients and caregivers towards psychotropic medications were associated with lower adherence whereas better doctor-patient communication was associated with higher adherence to medications.

## Highlights

• The study has tried to explore the factors influencing medication adherence among patients with mental disorders.
• Patient related variables, caregiver’s attitude towards medication and doctor patient communication have been explored.
• The study findings will help predicting medication non-adherence and its addressal can lead to better patient outcomes.

## 1 Introduction

Severe mental disorders such as schizophrenia and bipolar disorder, by virtue of their chronic nature, constitute the leading causes of years lived with disability.^1^ Medications are an essential part of the treatment of such mental disorders; both in the acute phase as well as in long–term management. Medication adherence is defined by World Health Organization (WHO) as “the degree to which the person's behavior corresponds with the agreed recommendations from a health care provider.^2^ Several studies have shown that the relapse rate is significantly lower with drug therapy, provided that the patient is adherent.^3^

Contemporary research shows that non-adherence to psychotropic medications varies around 28–52% of patients with depression; up to 50% among patients diagnosed with bipolar disorder, up to 70% among patients with schizophrenia, and 57% among patients with anxiety disorders.^4^ Further, literature reports that nearly 40% of patients stop taking their medication within a year and it's 75% by the end of two years.^5^

Medication non-adherence causes abrupt worsening of the illness; reduction in the effectiveness of the management and results in poor response to subsequent treatment.^6^ Further, medication non-adherence increases mortality and morbidity.^7^, ^8^, ^9^, ^10^

Studies from different geographical areas have described the factors for medication non-adherence such as poor insight, presence of positive symptoms, associated comorbidity, substance abuse, side effects of the drugs, and unemployment.^11^, ^12^, ^13^ In developing countries, non-adherence to medications has been attributed to poor socioeconomic status, poor family support, associated stigma, poor insight towards the illness, illiteracy, side effects, social and cultural myths, stigma, loss, and grief.^14^^,^^15^

Among the psychosocial factors, the negative attitude of patients' caregivers towards psychotropic medications has been found to adversely impact medication adherence.^16^ A positive attitude of patients towards medications was associated with better medication adherence in previous studies.^17^^,^^18^ Hence, evaluation of the attitude of patients and caregivers are warranted in assessment of factors influencing medication adherence.

Limited studies have evaluated the effects of patient-specific factors such as doctor-patient communication and stigma, on medication adherence among patients with severe mental disorders. The current study aimed to explore the association between medication non-adherence and several variables including socio-demographic, clinical factors, self-stigma, patients' and caregivers' attitudes towards psychotropic medication, and quality of doctor-patient communication.

## 2 Methods

The study was an observational, cross-sectional, hospital-based study conducted at the Department of Psychiatry of a tertiary health care centre in the southern part of India. The study was conducted over a period of 18 months, from May 2020 to November 2021.

By using convenience sampling method, patients aged 18 to 65 years of all genders, who attended outpatient unit with one or more of the following severe mental disorders according to Diagnostic and Statistical Manual of Mental disorders-5 (DSM-5) - schizophrenia, schizoaffective disorder, delusional disorder, bipolar disorder, and major depressive disorder - were screened for inclusion into the study. Patients with physical comorbidities such as chronic medical illness (diabetes mellitus, systemic hypertension, etc.) and substance use disorders, were also included in the study.

Those patients with major neurocognitive disorder and intellectual disability as per DSM-5 were excluded from the study. The patients with the above-mentioned disorders may find it cognitively difficult to complete the questionnaires which could provide misleading findings and were hence excluded from participation. Further, those patients presenting with agitation and who were uncooperative for the interview were also excluded. The caregivers with any mental disorder, neurocognitive disorders, and intellectual disability were excluded from participating in the study.

Informed consent from patients and caregivers was obtained. If the patient was unable to give valid consent, the consent was taken from the caregivers or legally appointed representative (LAR) of the patient. Subsequently, when the patient improved, consent was taken from the patient. The study was approved by the Institutional Human Ethics Committee (IHEC).

The patients were evaluated at the time of presentation irrespective of their clinical status either in the acute phase or in remission. The questionnaires were administered to patients and caregivers by one of the authors after explaining the study procedure and procuring informed consent. Socio-demographic data and clinical variables such as the diagnosis, number of episodes, and hospitalizations were recorded using a semi-structured data collection form. The severity of the patient's illness was rated using illness-specific rating scales - Brief Psychiatric Rating Scale (BPRS),^19^ Hamilton Depression Rating Scale (HAM - D),^20^ Young Mania Rating Scale (YMRS).^21^

The Medication Adherence Rating Scale (MARS) was used for assessing medication adherence among the patients.^22^ The MARS scale contains 10 (yes/no) items and the sum of items yields a final score ranging from 0 (poor adherence to treatment) to 10 (good adherence to treatment). Based on MARS score, individuals are classified as non-adherent (scores of 0 to 7) and adherent (scores of 8 to 10).^23^

The Internalised Stigma of Mental Illness Inventory - 9 (ISMI - 9) was used for assessing patients' self-stigma about mental illness.^24^ The ISMI - 9 contains 9 items pertaining to the patient's experience of stigma and is measured on a four-point Likert scale. A high total score on the ISMI scale indicates a more severe internalised stigmatization.^24^

Attitude towards psychotropic medication was assessed using patient and caregiver versions of the Attitude of patient and caregiver towards psychotropic medication scale.^25^ Out of the 18 items on the questionnaire, 10 items were used to assess negative attitudes; whereas 8 items were used to assess positive attitudes towards psychotropic medications. This questionnaire is a self-report measure with each item measured on a 3-point Likert scale.^25^

The Doctor-Patient Communication Questionnaire (DPCQ) was used to find out the patient's perception about the quality of interaction with the health care providers.^26^ The scale takes the patient's point of view when evaluating the doctor-patient communication pattern. It consists of 15 questions measured on a four-point Likert scale and the total score amounts to 60.^26^

The distribution of the variables was assessed for normality by the Kolmogorov-Smirnov test. Descriptive statistics such as mean and percentages were used to portray the distribution of socio-demographic variables, illness-related variables, MARS scores, ISMI-9 scores, Attitude of patient and caregiver scores, and DPCQ scores. Independent ‘t’ test, ANOVA, and chi-square test were used to compare the distribution of the above variables among the adherent and non-adherent groups. Correlation analysis between the variables in the adherence and non-adherence groups was carried out by Pearson's correlation. Multivariate logistic regression was utilized to predict the independent risk factors for medication adherence in the study sample. Data were analyzed using Statistical Package for Social Sciences (SPSS) software (IBM, Chicago, USA, version 21.0). A *p*-value of less than or equal to 0.05 was considered to be statistically significant.

## 3 Results

A total of 152 patient-caregiver pairs participated in this study. The mean age of the patients was 34.33 ± 11.3 years. The majority of the patients were females (63.16%), married (64.47%), and completed middle school and belonged to middle socioeconomic status (65.79%) and hailed from semi-urban backgrounds (42.11%) **(**Table 1**).**

**Table 1:** Distribution of sociodemographic variables among patients and caregivers.

**Age (years)**	34.33 ± 11.3 (Mean ± SD)
Gender	
Male	56 (36.84%)
Female	96 (63.16%)

Marital status	
Unmarried	48 (31.58%)
Married	98 (64.47%)
Separated	1 (0.66%)
Widow	5 (3.29%)

Educational status	
Profession	12 (7.89%)
Graduate	46 (30.26%)
Intermediate or diploma	4 (2.63%)
High school	18 (11.84%)
Middle school	42 (27.63%)
Primary school	25 (16.45%)
Illiterate	5 (3.29%)

Socio economic status	
Upper	30 (19.74%)
Upper - middle	48 (31.58%)
Lower - middle	52 (34.21%)
Upper - lower	21 (13.82%)
Lower	1 (0.66%)

Area of domicile	
Rural	39 (25.66%)
Semi - urban	64 (42.11%)
Urban	49 (32.24%)
Caregiver's age (years)	40.89 ± 11.38 (Mean ± SD)

Caregiver's gender	
Female	66 (43.42%)
Male	86 (56.58%)

Caregiver's educational status	
Professional	10 (6.58%)
Graduate	34 (22.37%)
Intermediate or diploma	7 (4.61%)
High school	17 (11.18%)
Middle school	45 (29.61%)
Primary school	28 (18.42%)
Illiterate	11 (7.24%)

The mean age of the patient's caregivers was 40.89 ± 11.38 years majority being. Males (56.58%), married (82.24%) **(**Table 1**).**

Patients with a diagnosis of schizophrenia spectrum and other psychotic disorders constituted 45.4% of the sample, while 54.6% had a mood disorder. The mean duration of illness was 45.54 ± 44.54 months. The mean number of episodes among the patients with episodic illness was 2.04 ± 1.61. The mean number of admissions/hospitalisations was 1.12 ± 1.37. The majority of the patients had no comorbid psychiatric diagnosis (76.32%) or any medical comorbidities (61.18%) **(**Table 2**).**

**Table 2:** Comparison of adherent and non-adherent patients with reference to psychiatric diagnosis and medical comorbidities.

Psychiatric diagnosis	Adherent patients (MARS score 8–10) (*n* = 18)	Non-adherent patients (MARS score 0–7) (*n* = 134)	P - value
Schizophrenia	6 (33.33%)	52 (38.81%)	0.953
Delusional disorder	1 (5.56%)	10 (7.46%)	
Bipolar disorder type 1	5 (27.78%)	27 (20.15%)	
Bipolar disorder type 2	2 (11.11%)	17 (12.69%)	
Major depressive disorder	4 (22.22%)	28 (20.90%)	

Medical comorbidities			
No	9 (50%)	84 (62.69%)	χ^2^ = 1.075 (1) p = 0.3
Yes	9 (50%)	50 (37.31%)	

The mean BPRS score was 48.60 ± 8.34 among patients with schizophrenia spectrum and other psychotic disorders. The mean score on HAM–D among the 58 patients with depression (both bipolar disorder type-2 and major depressive disorder) was 22.81 ± 7.35. The mean score on YMRS was 33.81 ± 8.51 among the 32 patients with bipolar disorder type 1.

The mean value of the Medication Adherence Rating Scale (MARS) score among the patient was 4.27 ± 2.52. Majority of the patients (88.16%) were categorised as non-adherent to medications, based on MARS score. The median value of Internalised Stigma of Mental Illness Inventory – 9 (ISMI – 9) scores among the patients was found to be 2.1. The mean value of the Doctor-Patient Communication Questionnaire (DPCQ) score among the patients was 57.22 ± 3.06. The median of positive scale score in the Attitude of the patient's towards psychotropic medications scale was 21, whereas negative scale score was 25. The attitude towards psychotropic medications was predominantly negative among majority the patients (76.97%). The median of positive scale score in the Attitude of the caregivers towards psychotropic medications scale was found to be 22, whereas negative scale score was 26. The attitude towards psychotropic medications was predominantly negative among majority of caregivers (83.55%).

MARS score was significantly higher among patients of upper-middle socioeconomic class **(*p* = 0.033)** and urban area of domicile **(*p* = 0.014) (**Table 3**)** The educational level was higher among patients with medication adherence compared to patients with non-medication adherence which approached statistical significance **(*p* = 0.058).** There was no significant difference between the adherent and non-adherent groups with respect to psychiatric diagnosis (*p* = 0.953) and medical comorbidities (*p* = 0.34) **(**Table 2**).** The age of the caregivers positively correlated with MARS score **(*p* = 0.019)** (Table 4).

**Table 3:** Association between sociodemographic variables and medication adherence (MARS score).

Sociodemographic variables	Categories	MARS score Mean ± SD	*t* - test (t) / ANOVA (F) (df)	P - value
Gender of patient	Female (*N* = 96)	4.44 ± 2.52	*t* = 1.077 (150)	0.283
	Male (*N* = 56)	3.98 ± 2.5		
Marital status of patient	Unmarried (*N* = 48)	4.83 ± 2.48	F = 1.399 (3)	0.245
	Married (*N* = 98)	4.03 ± 2.48		
	Separated (*N* = 1)	2 ± 0		
	Widow (*N* = 5)	4 ± 3.24		
Educational status of patient	Professional (*N* = 12)	4.5 ± 2.07	F = 1.695 (6)	0.126
	Graduate (*N* = 46)	5.07 ± 2.43		
	Intermediate or diploma (*N* = 4)	5.5 ± 3.11		
	High school (*N* = 18)	3.72 ± 2.72		
	Middle school (*N* = 42)	3.95 ± 2.63		
	Primary school (*N* = 25)	3.44 ± 1.96		
	Illiterate (*N* = 5)	4.2 ± 3.49		
Occupational status of patient	Senior officer / manager (*N* = 1)	4 ± 0	F = 0.314 (7)	0.947
	Professionals (*N* = 12)	4.33 ± 2.71		
	Skilled / sales workers (*N* = 9)	3.89 ± 2.47		
	Skilled agricultural / fishery (*N* = 9)	3.44 ± 2.74		
	Craft/trade worker (*N* = 1)	2 ± 0		
	Machine operators (*N* = 8)	4.25 ± 2.76		
	Elementary occupation (*N* = 91)	4.36 ± 2.43		
	Unemployed (*N* = 21)	4.48 ± 2.87		
Socioeconomic status	Upper (*N* = 30)	4.53 ± 2.58	F = 2.708 (4)	**0.033**⁎
	Upper middle (*N* = 48)	5 ± 2.32		
	Lower middle (*N* = 52)	3.96 ± 2.42		
	Upper lower (*N* = 21)	3.05 ± 2.71		
	Lower (*N* = 1)	3 ± 0		
Area of domicile	Rural (*N* = 39)	4.18 ± 2.77	F = 4.387 (2)	**0.014**⁎
	Semi urban (*N* = 64)	3.7 ± 2.43		
	Urban (*N* = 49)	5.08 ± 2.23		

⁎ p significant at ≤**0.05.**

**Table 4:** Correlation between variables and MARS score.

Variables	Pearson Correlation coefficient	95% Confidence interval	p - value
Caregivers' age and MARS score	0.19	[0.03176, 0.3389]	**0.019**⁎
BPRS score and MARS score	−0.456	[−0.57, −0.32]	**0.0001**⁎
HAM - D score and MARS score	−0.451	[−0.57, −0.31]	**0.0004**⁎
YMRS scores and MARS score	−0.566	[−0.67, −0.45]	**0.0007**⁎
ISMI - 9 score and MARS score	−0.393	[−0.52, −0.25]	**<0.0001**⁎
Attitude of caregivers towards psychotropic medications - negative scale scores and MARS score	−0.241	[−0.39, −0.085]	**0.003**⁎
DPCQ score and MARS score	0.38	[0.24, 0.51]	**<0.0001**⁎

⁎ p significant at ≤**0.05**; Medication Adherence Rating Scale (MARS); Brief Psychiatric Rating Scale (BPRS); Hamilton Depression Rating Scale (HAM - D); Young Mania Rating Scale (YMRS); Internalised Stigma of Mental Illness inventory - 9 (ISMI - 9); Doctor-patient communication questionnaire (DPCQ).

MARS scores negatively correlated with BPRS score **(*p* = 0.0001)** among patients with schizophrenia and delusional disorder. Similarly, a significant negative correlation was noted between MARS score and HAM-D scores **(*p* = 0.0004)** as well as YMRS scores (***p* = 0.0007)**, among patients with mood disorders. The YMRS score was significantly higher among patients of bipolar disorder type 1 with medication non-adherence, as compared to patients with medication adherence group **(*p* < 0.0001) (**Table 4**).**

The scores on ISMI–9 showed a negative correlation with MARS score **(*p* < 0.001)**. Similarly, a significant negative correlation was noted between the attitude of the caregivers towards psychotropic medications - negative scale scores and MARS score **(*p* = 0.003) (**Table 4**).**

The Attitude of caregivers towards psychotropic medications - negative scale score was significantly higher among caregivers of patients with medication non-adherence, as compared to caregivers of patients with medication adherence **(*p* = 0.038). (**Table 4**)** Further, the Attitude of patients' towards psychotropic medications - negative scale score was higher among patients with medication non-adherence compared to patients with medication adherence, in a trend towards statistical significance **(*p* = 0.052)**. The Doctor-patient communication questionnaire (DPCQ) score was significantly higher among patients with medication adherence, as compared to patients with medication non-adherence **(*p* < 0.0001**)**(**Table 4**).**

Multiple logistic regression was performed to identify independent risk factors for medication non-adherence. Based on independent *t*-tests the following scores were found to have a significant association with medication non-adherence: YMRS score, Doctor-patient communication questionnaire score, Attitude of caregivers towards psychotropic medications - negative scale scores. On performing multiple logistic regression, none of the aforementioned variables were found to be independent risk factors for medication non-adherence **(**Table 5**).**

**Table 5:** Multivariate logistic regression to find out independent risk factors of medication non-adherence.

Medication non - adherence	Beta coefficient	Standard Error	*P* value	Odds ratio	95% C.I. for odds ratio	
					Lower	Upper
YMRS scores	0.667	0.559	0.233	1.948	0.651	5.824
Doctor-patient communication questionnaire score	−0.687	0.442	0.120	0.503	0.212	1.195
Attitude of patient's Caregivers towards psychotropic medications- negative scale scores	0.293	0.236	0.214	1.340	0.844	2.126

## 4 Discussion

The current study is cross-sectional in nature where a total of 152 patients and caregivers who were attending out-patient psychiatric services in a tertiary care centre were taken up for the study. An attempt was made to evaluate the relationship between medication non-adherence in patients with severe mental disorders and various socio-demographic, clinical-factors, self-stigma, patients' and caregivers' attitudes towards psychotropic medications, and doctor-patient communication.

In this study, the proportion of patients with medication non-adherence was much larger than the proportion of patients with medication adherence. Medication non-adherence among patients is influenced by demographic factors such as socioeconomic status, area of domicile, and caregivers' age. Medication adherence score (MARS) was higher among those belonging to upper-middle socioeconomic status and urban areas of domicile. This finding is in concordance with a previous study which reported that upper middle socioeconomic status and urban people are more adherent to medications than rural populations.^27^, ^28^, ^29^ Higher age of caregiver was associated with better medications adherence similar to existing literature which revealed that elderly caregivers were able to understand the disease process in a better manner resulting in improved adherence rates, among their patients, compared to those patients cared by younger caregivers.^30^

Higher illness severity scores (BPRS, HAM-D, YMRS) were associated with medication non-adherence among patients with severe mental disorders. Medication non-adherence can lead to worsening of illness severity and which in turn can lead to poor insight into the illness leading to adverse clinical outcomes. However, this is in contrast to existing literature where no significant association between medication non-adherence rates and illness severity scores in participants with mood disorders.^31^^,^^32^ However, a significant association between medication non-adherence rates and illness severity scores in patients with schizophrenia has been observed.^33^

Higher ISMI-9 scores, indicating higher levels of self-stigma was associated with medication non-adherence. Higher levels of stigma can be associated with decreased willingness in seeking psychiatric services which in turn can lead to non-adherence and adverse outcomes in such patients.^34^ Existing literature emphasizes the impact of self-stigma on medication adherence by various researchers who have found that higher self-stigma scores are associated with medication non-adherence, which are similar to the current study findings.^35^, ^36^, ^37^

The Doctor-patient communication questionnaire (DPCQ) score was higher among patients with medication adherence, as compared to patients with medication non-adherence, implying that better doctor-patient communication was associated with better medication adherence. A similar study among patients with severe mental disorders reported that doctors communicating adequately with the patient regarding the illness can reduce non-adherence to medications.^4^^,^^38^, ^39^, ^40^ The presence of better communication between doctors and patients can possibly enhance the doctor-patient relationship and better health-seeking behaviors among patients with respect to illness severity, adverse drug events, and regular follow-ups cumulatively resulting in better medication adherence and ultimately better health outcomes. The negative attitude of patients and caregivers towards psychotropic medications were found to be associated with medication non-adherence, which is similar to the current study.^16^ A negative attitude towards medications, pertaining to adverse events or anticipated long-term outcomes, may reduce adherence to medications.

However, none of the above factors significantly predicted non-adherence to medications among patients with severe mental disorders.

Limitation**s** of the study were limited sample size with further limited participants in the medication adherent group. The utilization of self-report questionnaires may have an inherent recall bias by the participants, especially considering their clinical status during inclusion in the study. The potential confounding effect of the nature of medications and their adverse effects on medication adherence was not evaluated in the current study. The role of psychological interventions in enhancing adherence has not been studied in this sample as the study design was cross-sectional in nature.

## 5 Conclusion

Medication non-adherence is a significant problem in the management of patients with severe mental disorders. Medication adherence was higher among patients belonging to upper-middle socioeconomic status, urban domicile, and higher caregiver age. Higher severity of illness, self-stigma, and negative attitude of patients and caregivers towards psychotropic medications were associated with lower adherence whereas better doctor-patient communication was associated with better adherence. A follow-up study design analysing the pattern of medication adherence over various phases of the illness and a qualitative analysis of the knowledge, attitude, and perception of the patients and caregivers towards psychotropic medications could provide more insights. This, in turn, can improve the health outcome of patients with severe mental disorders.

## Declaration of Competing Interest

The authors declare that they have no known competing financial interests or personal relationships that could have appeared to influence the work reported in this paper.
